# Magnetic Micromachine Using Nickel Nanoparticles for Propelling and Releasing in Indirect Assembly of Cell-Laden Micromodules

**DOI:** 10.3390/mi10060370

**Published:** 2019-06-01

**Authors:** Jianing Li, Huaping Wang, Juan Cui, Qing Shi, Zhiqiang Zheng, Tao Sun, Qiang Huang, Toshio Fukuda

**Affiliations:** Intelligent Robotics Institute, School of Mechatronical Engineering, Beijing Institute of Technology, 5 South Zhongguancun Street, Haidian District, Beijing 100081, China; 2120170179@bit.edu.cn (J.L.); cuijuan2016@bit.edu.cn (J.C.); shiqing@bit.edu.cn (Q.S.); zzqyouyou@sina.com (Z.Z.); 3120120061@bit.edu.cn (T.S.); qhuang@bit.edu.cn (Q.H.); tofukuda@nifty.com (T.F.)

**Keywords:** magnetic manipulation, magnetic nanoparticles, bioassembly, tissue engineering

## Abstract

Magnetic micromachines as wireless end-effectors have been widely applied for drug discovery and regenerative medicine. Yet, the magnetic assembly of arbitrarily shaped cellular microstructures with high efficiency and flexibility still remains a big challenge. Here, a novel clamp-shape micromachine using magnetic nanoparticles was developed for the indirect untethered bioassembly. With a multi-layer template, the nickel nanoparticles were mixed with polydimethylsiloxane (PDMS) for mold replication of the micromachine with a high-resolution and permeability. To actuate the micromachine with a high flexibility and large scalable operation range, a multi-pole electromagnetic system was set up to generate a three-dimensional magnetic field in a large workspace. Through designing a series of flexible translations and rotations with a velocity of 15mm/s and 3 Hz, the micromachine realized the propel-and-throw strategy to overcome the inevitable adhesion during bioassembly. The hydrogel microstructures loaded with different types of cells or the bioactive materials were effectively assembled into microtissues with reconfigurable shape and composition. The results indicate that indirect magnetic manipulation can perform an efficient and versatile bioassembly of cellular micromodules, which is promising for drug trials and modular tissue engineering.

## 1. Introduction

The fabrication and manipulation of micro/nanostructure play an important role in various applications including microelectronic devices and biomedical research [1,2,3]. For clinical diagnosis and treatment, magnetic micromachine can work in a confined space flexibly and has been applied for capsule endoscope [4,5] and minimally invasive surgery [6,7]. Since magnetic force is primarily exerted on magnetic substances, it is crucial to design and implement complex micromachine with desired shape and composition for specific tasks [8,9,10]. Magnetic nanoparticles (MNPs), as the superparamagnetic nanomaterial with controllable sizes ranging from a cell dimension to a virus dimension, provide the ability to coat or bond with biological entities for wireless transport and immobilization tasks under microscale [11,12]. Through compounding MNPs with biocompatible or biodegradable polymers, the magnet-tagged micromachines can be fabricated as microcarrier or end-effector for versatile, dynamic, and accurate biomanipulation [13,14]. Therefore, the development of MNPs-based micromachine with customized architecture and control scheme is meaningful in pharmacological research and tissue engineering.

A lot of magnetic microcarriers have been reported which can load drugs, cells, or other bioactive materials for biological research including drug discovery and regenerative medicine. Inspired by microbes, Nelson’s group developed a series of deformable microrobots with flagella, adapted to different complex environments [15,16]. Li et al. invented a symmetric multilinked two-arm nanoswimmer, providing powerful propulsion up to twelve body lengths per second [17]. Lu et al. proposed a multi-legged soft millirobot, which shows superior carrying capacity and obstacle-crossing performance in both dry and wet conditions [18]. Although these micromachines exhibit desirable mobility, most of them were designed for specific therapies and need to manually load the target material into the body in advance. It is not facile enough for bioassembly which needs to carry arbitrarily shaped microstructures. Furthermore, the direct contact between the MNPs and bio-target still risk the potential cytotoxicity of heavy metal [19,20,21]. More flexible and biocompatible magnetic manipulation strategies for the assembly of a biological target still remain lacking.

To overcome the above problems, an effective approach is to utilize magnetic micromachine as an intermediate (i.e., an end-effector) for indirect manipulation of biostructures with no MNP [22]. Based on this concept, Sitti described a method that the NdFeB microrobot (so-called Mag-μBot) was used to push and assemble various microgels with tunable shapes for bottom-up tissue engineering [23]. Diller proposed two kinds of magnetic micro-grippers, opened or closed by a magnetic field to capture or release microstructure [24,25]. Huang reported a compound micro-transporter consisting of a magnetic screw and a cylindrical bushing, which can actively collect, transport, and controllably release micro- and nanoagents [26]. However, as a wireless end-effector, the micromachines need complex control scheme to realize the grip-and-release strategies, which result in a trade-off for the limited performance of mobility and workspace. Even with the sophisticated design, the magnetic microgripper still can only assemble one type of specifically shaped microstructure in a narrow area. It is not suitable for large-scale bioassembly tasks which need to be accomplished in limited time for cell viability maintenance and structure scaling up. Overall, to assemble arbitrary cellular microstructures on a large scale, an efficient and versatile strategy for indirect magnetic manipulation under a high magnetic field is still required.

In this paper, we propose a novel clamp-shape micromachine with a propel-and-throw strategy for indirect assembly of cell-laden micromodules. Compared with other MNPs, nickel nanoparticles were chosen as magnetic substances due to high magnetic permeability and relatively unchanged properties. Combining nickel nanoparticles and polydimethylsiloxane (PDMS), the micromachine was fabricated by mold replication. A multilayer mold was designed to achieve desired shapes with 10-μm resolution. To actuate the micromachine with high flexibility and large scalable operation range, an eight-pole electromagnetic system with a cube-vertex distribution was set up to generate a 3D magnetic field and gradient up to 80 mT and 1.6 T/m. The modeling and control algorithm was designed for various rotations and translations of the micromachine, which realized the propelling and throwing strategies to overcome the inevitable adhesion between the assembly target and the micromachine. The cell-laden micromodules were photo-crosslinked with PEGDA hydrogel and shaped into customized structures. With the propel-and-throw micromanipulation, the micromodules carrying cells or bioactive materials were assembled into microtissues with reconfigurable structure and composition. The results demonstrate that the micromachine had precise mobility and flexible manipulability, which provided a prospective method for modular tissue engineering and local interaction study between drugs and cells.

## 2. Materials and Methods

### 2.1. Fabrication of Magnetic Micromachines

**Figure 1:**
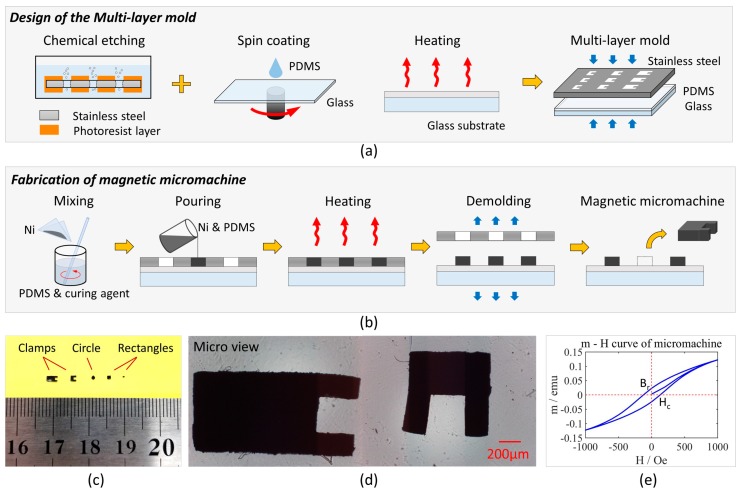
(**a**) Design of the multi-layer mold. Through-hole patterns were etched on the stainless steel template. It was matched with the glass substrate coated a polydimethylsiloxane (PDMS) layer to form a multi-layer mold. (**b**) Fabrication of magnetic micromachines. Nickel nanoparticles were mixed with PDMS and poured into the mold. After heating and demolding, the magnetic micromachine peeled off. (**c**) Macro view of micromachines. Apart from the clamp shape, circles and rectangles were designed, ranging from 2000 μm × 1000 μm × 300 μm to 300 μm × 300 μm × 300 μm. (**d**) Micro view of micromachines with the clamp shape. (**e**) Hysteresis (magnetic moment-external field) of a micromachine measured by vibrating sample magnetometer (VSM).

Mold replication is a batch method for the fabrication of magnetic micromachines [27,28]. As shown in Figure 1a, a multi-layer mold was designed to improve precision and repeatability. The stainless steel template had high stiffness and ccould be reused without wear, compared with conventional PDMS or SU-8 soft-lithography [29,30]. Chemical etching is a common method to pattern stainless steel with high-resolution, which determines the shape and height of micromachines [31]. Through-hole patterns with symmetrical sidewall were formed to ensure the stability of micromachine during the indirect propulsion. To match the stainless steel template, a glass substrate was coated with a PDMS layer for seamless adhesion. PDMS was mixed with the curing agent at a mass ratio of 10:1. Then, the mixed solution was poured on the glass substrate to form a 50-μm layer by spin coating at 1000 rpm for 30 s. After baking at 60 °C for 4 h, the glass substrate was matched with the through-hole template to form an entire mold.

For fabrication of magnetic micromachines, nickel nanoparticles were chosen as the magnetic substance. The diameter of nickel nanoparticles is suitable for precise microfabrication, while permanent magnets (e.g., NdFeB) is limited by their domains. Compared with conventional ferrite materials, nickel nanoparticles show high magnetic permeability and relatively unchanged properties even from different processes, which is suitable for repeatable experiments. The procedure is shown in Figure 1b. First, the nickel nanoparticles and PDMS solution (10%w/w curing agent) were mixed at a mass ratio of 1:1. A series of ratios were tested and the results were shown in Supplementary Figure S1. Then, the mixture was poured into the multi-layer mold. After removing redundant mixture and bubbles, it was baked at 60 °C for 6 h. Since both the substrate and micromachines contained PDMS, the compatibility ensured the bottom precision of micromachines. Due to curing in different steps, they were easily separated. Finally, the intact micromachines were taken out by splitting the template and the substrate. Different micromachines were designed from 300 μm × 300 μm × 300 μm to 2000 μm × 1000 μm × 300 μm. As shown in Figure 1e, the micromachines show a high-resolution of 10 μm. The magnetization measured by vibrating sample magnetometer (VSM) exhibited high permeability and hysteresis loop, consistent with the former references.

### 2.2. Fabrication of Micromodules

**Figure 2:**
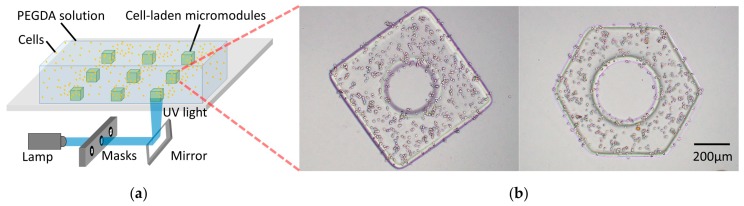
(**a**) Fabrication of cell-laden micromodules. (**b**) Optical images of two micromodules.

For indirect assembly, a lot of cell-laden micromodules were fabricated by shape-controllable photo-crosslinking. NIH/3T3 fibroblast cells were cultured and trypsinized to obtain a 5 × 10^7^ cells mL^−1^ suspension. The cell suspension was mixed with poly(ethylene glycol) diacrylate (PEGDA) and PI as a prepolymer solution. Particularly, PEGDA was modified by Arg-Gly-Asp-Ser (RGDs) to improve cell adhesion and viability. The specific procedure was introduced in References [32,33]. The fabrication of micromodules is shown in Figure 2. To achieve a controllable light pattern, the ultraviolet (UV) light was generated by a mercury lamp and passed through a mask with a predesigned pattern. Under UV irradiation, a PEDGA prepolymer solution was cross-linked into hydrogel micromodules with the 2D desired shape, where the cells were encapsulated. The planar pattern of the micromodules can be flexibly changed by switching the masks. The photo-crosslinking process was conducted in the microfluidic channel, which determines the height of micromodules.

## 3. External Magnetic Actuation

### 3.1. Electromagnetic System

**Figure 3:**
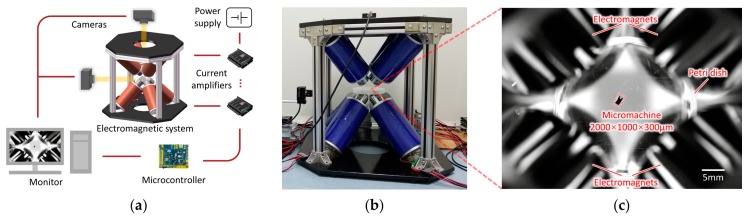
(**a**) Schematic of the external electromagnetic system. (**b**) Photograph of the eight-coil system. (**c**) Image of the micromachine in the workspace from the top camera.

To actuate the magnetic micromachine, a multipole electromagnetic system (shown in Figure 3) was set up to generate a 3D magnetic field and gradient. Eight coils were distributed at each vertex of a cube and aligned with the center. The system had a spherical workspace with a diameter of 100 mm, enough to hold a 65 mm Petri dish. The maximum magnetic field and gradient near the workspace center reached 80 mT and 1.6 T/m. Each coil had 1150 turns of 1.8 mm copper wire. The inner diameter, outer diameter and length were 56 mm, 114 mm and 220 mm, respectively. The DT4 core had a length of 270 mm and its maximum permeability was 6000 H/m. Each electromagnet had a resistance of 5 Ω and an inductance of 60 mH. When the current was 1 A, one coil produced a magnetic field of approximately 4 mT in the workspace center.

Eight current amplifiers (ESCON 50/5, MAXON, Sachseln, Switzerland) were applied to provide programmable currents with the ±5 A range and 12-bit resolution. Each of them was connected to a 350 W power supply (LRS-350-48, MEANWELL, Guangzhou, China). Due to the inductive effect of coils, high power was necessary for the dynamic response of magnetic generation with control compensators. Two microcontrollers sent 16-bit resolution signals to the amplifiers and communicated with the computer at 50 Hz. User interface based on C# program was developed on the computer with a Win10 operating system. Two groups of cameras and lenses were mounted orthogonally for the top and side visual feedback. Each camera (MD028MU-SY, XIMEA, Münster, Germany) output images of 1934 × 1456 pixels with a resolution of 4.54 um. The lens (FA2502D, CHIOPT, Guangzhou, China) had a focus of 25 mm and a view angle of 20° × 15° × 24.6°. The data acquisition card was installed on the computer to transfer and record two channels of 16-fps grayscale videos at the same time.

### 3.2. Principe of Magnetic Actuation

For the electromagnetic system, the workspace was regarded as a free space, where there was no free charge or current. Since the object is much smaller than the workspace, the magnetic torque and force exerted on the object are given by

(1) $$T_{m}=m\times B$$

(2) $$F_{m}=(m\cdot \nabla )B$$

where ***m*** is the magnetic moment of the object, ***B*** is the magnetic field generated by the external system and ▽ is the gradient operator. In general, the magnetic torque is related to the magnetic field, while the magnetic force is related to the magnetic gradient. The micromachine used in this paper had obvious remanence and coercivity, resulting in a relatively constant magnetic moment after magnetization.

Magnetic torque caused the object’s rotation. If only the magnetic torque was considered, the stationary condition of the object was T = 0. Therefore, the magnetic moment must be parallel to the magnetic field. Since it was unstable for the opposite direction, the magnetic moment usually had the same direction as the magnetic field. It can be expressed as

(3) $$m=\left|m\right|\hat{B}$$

where | | represents the norm of vector and ^ represents the unit vector along the direction. It is noted that the magnetic torque has no effect on the rotation along the magnetic moment. In practice, it was avoided to magnetize the object along the axis of the desired rotation.

The magnetic force caused the object’s translation. If only the magnetic field was considered, the stationary condition of the object was *F* = 0. There is

(4) $$\begin{array}{cc} m\cdot \nabla B_{i}=0, & i=x,y,z \end{array}.$$

The solution contained local maxima and minima. Since minima were unstable cases, the object eventually stayed at the point where the magnetic field was a local maximum.

Considering other forces, the drag force was the main resistance. When the object moved at a low velocity, the drag force was given by

(5) $$F_{d}=6\pi \eta rv$$

where *η*, *r* and *v* are the fluid’s viscosity, the object’s radius and velocity, respectively. These parameters except the velocity were replaced by a measured ratio *k*. Newton’s second law is expressed as

(6) $$F_{m}-kv=ma.$$

If the magnetic force did not change with time, the object’s velocity was

(7) $$v=\frac{F_{m}}{k}(1-e^{-\frac{k}{m}t})+v_{0}e^{-\frac{k}{m}t}.$$

The first term represents steady state and the second term represents the transient state. In the beginning, the object had maximum acceleration. While the velocity and drag force increase, the acceleration decreases. When the forces achieved balance, the object had the maximum velocity, which was proportional to the magnetic force. The analysis of the motion state depended on the transient time, which is related to the term of k/m.

### 3.3. Magnetic Field Generation and Control Model

For the multi-coil system, the key issue was to calculate all currents needed for magnetic field generation. According to the Biot-Savart law, the magnetic field at position *r* generated by a current *I* is

(8) $$B=\frac{\mu_{0}}{4\pi }\int_{L}\frac{IdL\times \hat{r}}{|r{|}^{2}}$$

where *μ*_0_ is the vacuum permeability and *L* is the length vector of the wire. For electromagnetic coils, the general formula at an arbitrary position can only be expressed in integral form. For intuitive expression, the magnetic field along the cylindrical axis is given by

(9) $$B=\frac{\mu_{0}NI}{4\pi }\left[\frac{0.5L-x}{\sqrt{{\left(0.5L-x\right)}^{2}+R^{2}}}+\frac{0.5L+x}{\sqrt{{\left(0.5L+x\right)}^{2}+R^{2}}}\right]$$

where *N*, *I*, *L* and *R* are the turns, current, length and radius of the coil, respectively. It indicates that the magnetic field can be expressed as a function of position times the current, that is

(10) B=f(x,y,z)I.

If the function is regarded as a constant for a certain position, the magnetic field of one coil can be expressed by the current. For the eight coils in this paper, the magnetic field in the workspace can be derived from the transformation matrix.

(11) $$\left[\begin{array}{c} B_{x}\\ B_{y}\\ B_{z} \end{array}\right]=RfI=\frac{1}{\sqrt{3}}\left[\begin{array}{cccccccc} 1 & -1 & -1 & 1 & 1 & -1 & -1 & 1\\ 1 & 1 & -1 & -1 & 1 & 1 & -1 & -1\\ 1 & 1 & 1 & 1 & -1 & -1 & -1 & -1 \end{array}\right]\left[\begin{array}{ccc} f_{1} & & 0\\ & \ddots & \\ 0 & & f_{8} \end{array}\right]\left[\begin{array}{c} I_{1}\\ \vdots \\ I_{8} \end{array}\right].$$

In addition, the gradient of the magnetic field is related to the magnetic force. For the magnetic field and its gradient, all six values are determined by the eight currents.

**Figure 4:**
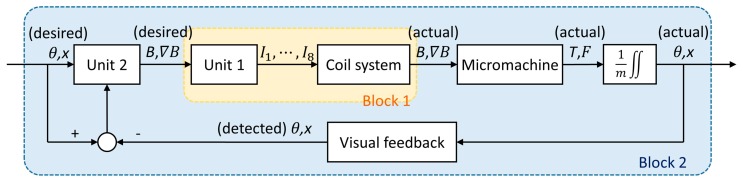
Position and direction control of the micromachine by magnetic actuation.

Based on the above concept, the motion control of magnetic actuation was modeled. The modeling of the whole system is shown in Figure 4. Unit 1 calculates all currents of the coil system to generate the target magnetic field distribution. For a good dynamic response, the equivalent value of Block 1 was close to one, so Unit 1 was designed based on the pseudoinverse matrix of the coil system. Unit 2 decides the required magnetic field distribution, which directly determines the final position and direction of the micromachine (discussed in Section 3.2). Therefore, the design of Unit 2 was mainly based on the target position and direction, while their deviations are a supplement. The expected value of Block 2 was also close to one, but it usually took a few seconds for a dynamic response.

**Figure 5:**
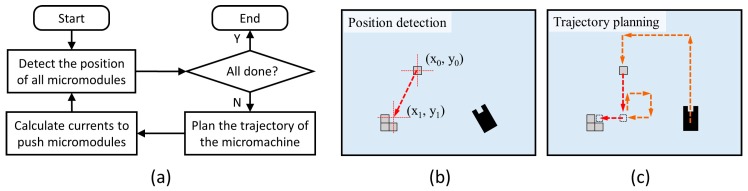
(**a**) Flow chart of micromodules’ assembly. (**b**) Diagram of one micromodule’s position detection. (**c**) Diagram of the micromachine’s trajectory planning.

For indirect assembly of micromodules, a cyclical manipulation strategy was adopted. The flow chart is shown in Figure 5. At the beginning of the loop, the position of all micromodules was detected by the visual feedback. As an isolated micromodule was detected, its destination and path were analyzed. Then, the micromachine’s trajectory was planned for the micromodule’s propulsion. Based on the required magnetic field, the trajectory was usually divided into several segments. For the motion of each segment, all currents were calculated by control units. The loop was repeated until all micromodules were located in the correct position.

## 4. Results and Discussion

### 4.1. Motion Measurement

**Figure 6:**
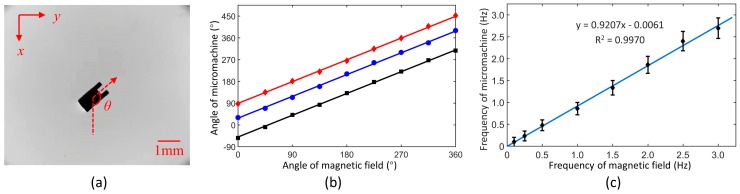
(**a**) Original image and angle definition of the micromachine under the magnetic field. (**b**) Angle relation of micromachines to the magnetic field. (**c**) Dynamic response of the micromachine’s rotation under the rotate magnetic field.

To verify the driving principle, the direction and position of micromachine under the magnetic field were recorded. To evaluate the mobility, rotation and translation were measured. For the micromachine’s direction in steady state, eight certain angles of the magnetic field were chosen as inputs. The angle definition of micromachine is shown in Figure 6a. Represented by the groove, the micromachine’s angle was measured five times for each field angle. To avoid the shape’s influence, three micromachines magnetized in different directions were tested individually. In the experiment, micromachines were placed on the liquid surface to reduce friction and improve precision. direction measurement is performed by image processing. The micromachine’s contour was extracted by Gaussian smoothing and Canny edge detection. The line’s slope was calculated by Hough transform. The angle uncertainty was no more than 2°.

As shown in Figure 6b, the result indicates that the included angle between the micromachine and the magnetic field was constant. As mentioned above, it was essential that the magnetic moment was always in the same direction as the magnetic field. Since the magnetic field was no more than the coercivity, the magnetic moment of every micromachine was almost unchanged during the experiment. Therefore, the magnetized angle of micromachines can be speculated in this way. It is noted that even if a constant magnetic field was input, the micromachine gradually moved to the edge of workspace due to imperfect uniformity. This could be avoided by reducing or removing the magnetic field in time. A better approach is to perform closed-loop control based on visual feedback.

To analyze the dynamic response of rotation, a series of rotational magnetic fields with a constant frequency was given in a range from 0 to 3 Hz. By counting rotational turns in 1 min, the average frequency of micromachine was calculated. The results are shown in Figure 6c. The micromachine’s rotation increased linearly with the frequency of the magnetic field, exhibiting a ratio of 0.9207. The actual value was closer to 1, considering the latency of the Windows system. The measurements of high frequency required a high-speed camera. Since it was unnecessary in our manipulation, the rotation at over 3 Hz has not been tested. In the subsequent experiments, it is believed that the micromachine remained in the same direction as the magnetic field in real time, as long as there was no high-frequency or long-time rotation.

**Figure 7:**
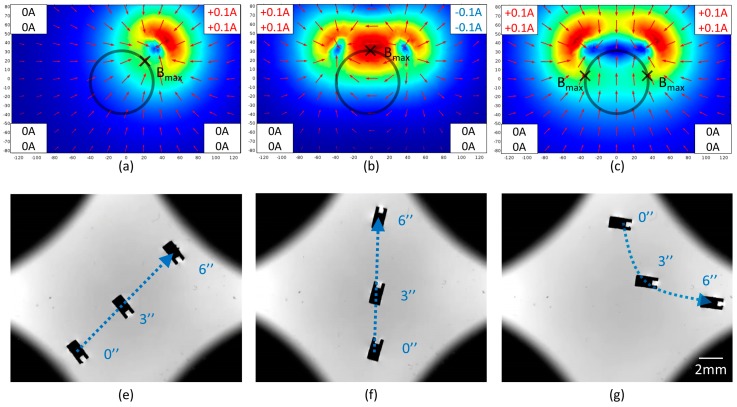
(**a**–**c**) Simulation of magnetic field distribution in three cases. The color and the arrow represent the magnitude and direction of the magnetic field respectively. The workspace and local maximum were marked by circles and crosses individually. (**e**–**g**) Images of the micromachine’s trajectory in three cases. The micromachine was magnetized at 90°.

For the micromachine’s position in a steady state, three cases were analyzed to conclude the general principle of position control. In the first case, only one group of up and bottom coils was working. Two groups were given opposite currents in the second case, while they were given the same currents in the third case. The simulations and trajectories are shown in Figure 7. As mentioned in Section 3.2, the micromachine moved toward the maximum magnetic field, meanwhile, its moment was parallel to the magnetic field. In the first case, there was no doubt that the micromachine moved toward the activated coils. In the second and third cases, the same number of coils were enabled. However, the micromachine moved toward the activated coils in the second case but backward in the third case. In Figure 7b, the red region in the workspace acted like a potential well and attracts the micromachine to the maximum. In Figure 7c, the blue region in the workspace acted like a potential barrier and pushed the micromachine to the left or right maximum. In the first and second case, the included angle between the micromachine and the magnetic field was different. In a subsequent experiment, the currents were configured based on the second case to generate an attractive force. Sometimes, the first case was applied to move sideways.

**Figure 8:**
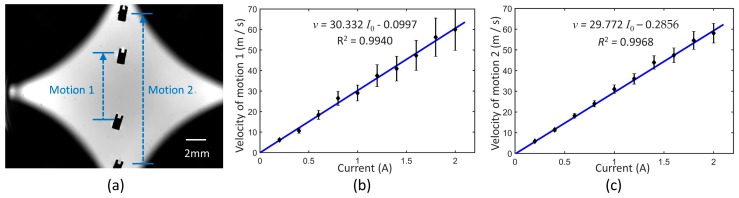
(**a**) Schematic of velocity measurement. (**b**) Average velocity of motion 1 actuated by coils. (**c**) Average velocity of motion 2 actuated by coils.

To analyze the dynamic response of translation, the system was given an equivalent current of 0–2 A to generate the magnetic gradient. Considering the non-uniformity of the magnetic gradient, two kinds of paths were measured to calculate the average velocity individually. Both of them started from the stationary state. The paths and results are shown in Figure 8. It indicates that the velocity was almost linear to the current when the current is no more than 2 A. When the micromachine began to move, the drag force increased and counterbalanced the magnetic force in a short time. Although the gradient in the workspace was not completely uniform, it had little influence on the linear relation. Since high-velocity limits control precision, the current was usually no more than 0.5 A in subsequent manipulation.

### 4.2. Propulsion by Magnetic Micromachine

**Figure 9:**
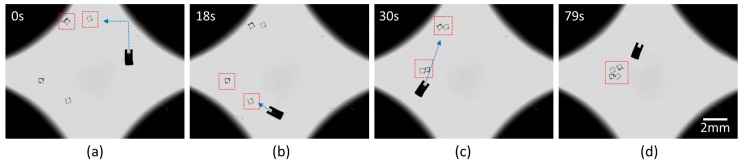
Snapshots of micromodules’ assembly by magnetic micromachine. (**a**) Assembly of the first subgroup; (**b**) assembly of the second subgroup; (**c**) assembly of the larger group; (**d**) images of the final entirety.

As previously mentioned, the whole bioassembly process based on propel-and-throw strategy was performed on the liquid surface to decrease the friction between the substrate and the micromachine. Since most of the cellular micromodules fabricated from hydrogel were hydrophilic, the micromachine and the assembly targets exposed in an environment with a multiphase (i.e., liquid and gaseous phases) wew strongly influenced by the scaling force. Thus, the propel-and-throw strategy not only simplified the tedious gripping motion but also avoided unnecessary adhesion. Referring to Figure 9, four micromodules as the targets were propelled to the same destination. To obtain a stable and accurate motion, the micromachine’s trajectory was separated into horizontal and vertical motions. According to the optimal path, every two micromodules were assembled into a subgroup. Then, two subgroups were integrated into a large group. The series of propulsion demonstrates high controllability and effectiveness. Due to the adhesion force at the microscale, these micromodules eventually gathered as an entirety.

**Figure 10:**
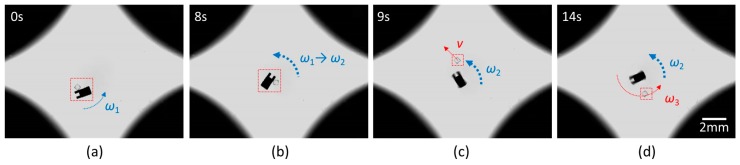
Snapshots of micromodules’ release by high-frequency rotation. (**a**) Angle adjustment of the entirety. (**b**) Increase of the rotational frequency. (**c**) Fly out of the micromodule along the tangent. (**d**) Rotation of the released micromodule caused by the vortex.

During the manipulation, the micromodules might adhere to the micromachine, which hinders the release of micromodules. One approach is to ensure the micromachine’s groove is aligned with the micromodule, which forms a laminar flow between them to avoid adhesion. Nevertheless, the position control is not always perfect and a small deviation will cause adhesion. Therefore, a throwing strategy (shown in Figure 10) was considered to release micromodules. At first, a low-frequency rotational magnetic field was given to make the micromachine and the micromodule rotate together. In this process, an appropriate angle was considered for release. Then, the field frequency increased suddenly and the micromodule was thrown out along the tangent line. Due to the drag force, the separated micromodule was supposed to stop in a short time. In practice, it’s observed that the released micromodule moves with the vortex flow generated by the high-velocity rotation of the micromachine. It’s a good choice to adjust the micromodule’s position by the vortex. The results show a good success rate to release the micromodules with a high positioning accuracy.

### 4.3. Bioassembly and Cultivation

**Figure 11:**
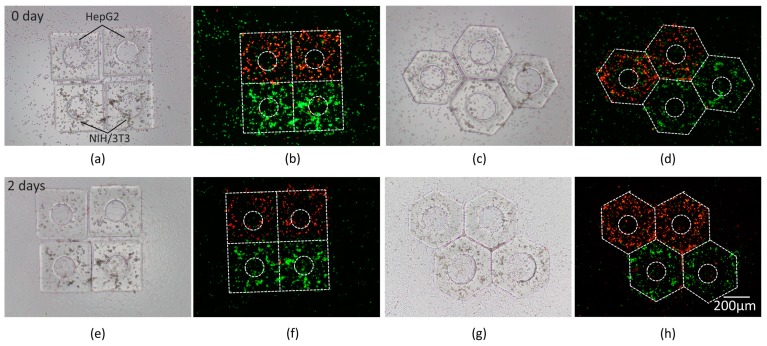
Co-culture of HepG2 and NIH/3T3 cells in micromodules; (**a**,**c**) Bright field images. (**b**,**d**) Fluorescence images. (**e**–**h**) Images after 2-day culture. HepG2 cells were labeled by a red fluorescent reagent and NIH/3T3 cells were labeled by a green fluorescent reagent. The contours of micromodules are marked by white dash lines.

To verify the biomedical application of the magnetic assembly, we performed the assembly of micromodules with different types of cells for co-culture and micromodules with bioactive materials which would be meaningful for drug evaluation in a local area. HepG2 and NIH/3T3 cells were encapsulated in PEGDA micromodules individually and assembled into an entirety for co-culture. Squares and hexagons were chosen for planar tessellation. To mimic the veins in microtissue, a hole in the center of micromodule was designed to promote nutrient diffusion and cell metabolism. After magnetic assembly, the solution was absorbed to make the micromodules adhere to the bottom surface of the dish. Then, the micromodules were placed in the CO_2_ incubator. Two kinds of cells were differentiable through fluorescence observation. After 2-day culture, cell proliferation on the surface was observed. As shown in Figure 11, a high level of cell viability demonstrates the biocompatibility of indirect magnetic manipulation.

**Figure 12:**
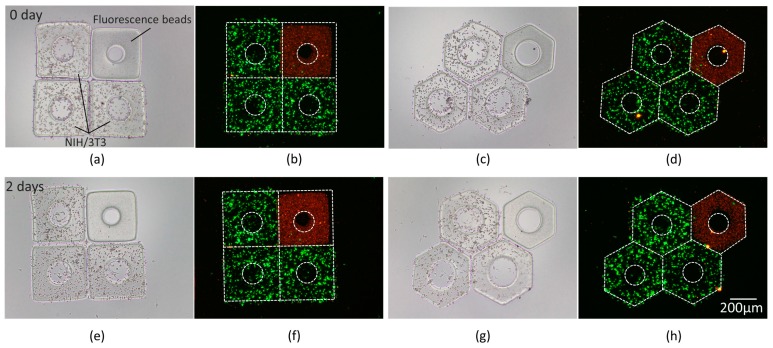
Images of red fluorescence beads and NIH/3T3 cells in micromodules; (**a**,**c**) Bright field images. (**b**,**d**) Fluorescence images. (**e**–**h**) Images after 2-day culture. The contours of micromodules are marked by white dash lines.

To demonstrate the application for local interaction between the target cells and drug, red fluorescence beads were encapsulated in micromodules and assembled with the micromodules containing NIH/3T3 cells. As shown in Figure 12, the cell viability and bead distribution were revealed by bright field and fluorescence observation. The engineered in-vitro tissues were usually regarded as a cellular model to study the cell behaviors influenced by different environmental parameters [34,35,36]. This experiment indicates an effective approach to assemble different cell-laden micromodules and load different drugs, which shows huge potential for drug trials. The proof-of-concept experiment of drug trials is clarified in Supplementary Materials as Figure S2.

## 5. Conclusions

This paper proposed an indirect untethered bioassembly method with a magnetic micromachine. Using nickel nanoparticles, the micromachine was fabricated by mold replication with a high-resolution and permeability. Actuated by an external multi-pole electromagnetic field, the micromachine exhibited flexible translation and rotation with a velocity up to 15 mm/s and 3 Hz, resulting in a precise position and direction. With the propel-and-throw strategy, the micromachine can effectively assemble cell-laden micromodules into reconfigurable tissue model. The results show that the micromachine was able to provide a sufficient force and effective assembly. In the future, a dynamic closed-loop control based on visual feedback will be considered to achieve three-dimensional magnetic actuation. With good mobility and biocompatibility, indirect magnetic manipulation provides a promising approach for bioassembly and tissue reconstruction.

## Supplementary Materials

The following are available online at https://www.mdpi.com/2072-666X/10/6/370/s1, Figure S1: Relation of the micromachine magnetization to the mass ratio of nickel nanoparticles, Figure S2: Schematic of proof-of-concept experiment for drug trials.
