# Supplementary material for: Magnetic Micromachine Using Nickel Nanoparticles for Propelling and Releasing in Indirect Assembly of Cell-Laden Micromodules
Source: Micromachines (Basel). 2019 Jun 1;10(6):370. doi: 10.3390/mi10060370 (PMC6631220; doi:10.3390/mi10060370)
Supplement: Supplementary file 1 [file micromachines-10-00370-s001.pdf]

micromachines

Article

Magnetic Micromachine Using Nickel Nanoparticles for Propelling and Releasing in Indirect Assembly of Cell-Laden Micromodules

Jianing Li, Huaping Wang *, Juan Cui, Qing Shi, Zhiqiang Zheng, Tao Sun, Qiang Huang and Toshio Fukuda

Intelligent Robotics Institute, School of Mechatronical Engineering, Beijing Institute of Technology, 5 South Zhongguancun Street, Haidian District, Beijing 100081, China; 2120170179@bit.edu.cn (J.L.); cuijuan2016@bit.edu.cn (J.C.); shiqing@bit.edu.cn (Q.S.); zzqyouyou@sina.com (Z.Z.); 3120120061@bit.edu.cn (T.S.); qhuang@bit.edu.cn (Q.H.); tofukuda@nifty.com (T.F.)

* Correspondence: wanghuaping@bit.edu.cn; Tel.: +86-68917765

Supplementary Figures

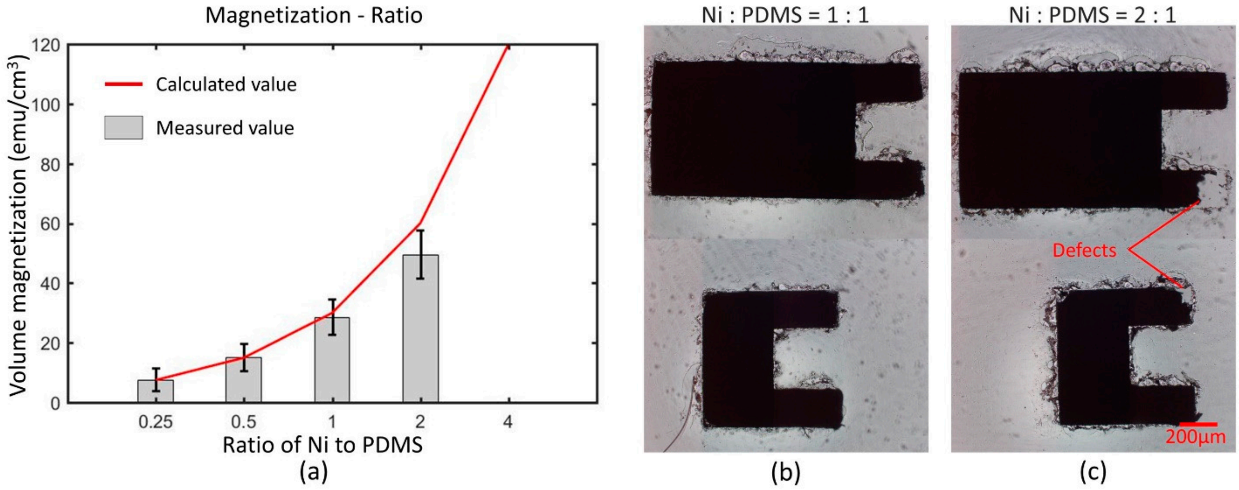

Figure S1. (**a**) Relation of the micromachine magnetization to the mass ratio of nickel nanoparticles. (**b**) Optical images of magnetic micromachine without demolding at the mass ratio of 1:1. (**c**) Optical images of magnetic micromachine at the mass ratio of 2:1. The defects of micromachines were marked.

As we know, the magnetic moment of micromachine is related to the mass ratio of magnetic substances. The magnetic micromachine in our paper consisted of nickel nanoparticles (NPs) and polydimethylsiloxane (PDMS). Since micromachines from different ratios have the same volume, we used the value K of NP mass m_{Ni} divided by the total volume V_{Total} to evaluate the magnetic moment. That is

$$K = \frac{m_{Ni}}{V_{Total}} . \quad (1)$$

It was observed that the total volume was only a little larger than the PDMS volume as long as NPs were not too much.

$$K = \frac{m_{Ni}}{V_{Total}} \approx \frac{m_{Ni}}{V_{PDMS}} = \rho_{PDMS} \frac{m_{Ni}}{m_{PDMS}}. \quad (2)$$

Therefore, the magnetic moment was almost proportional to the ratio of NPs to PDMS. To exert sufficient force, it was expected that the ratio was as large as possible. A series of ratios were investigated and the results is shown in Figure S2. When the ratio increased to 2:1, the poor fluidity of the mixture made it difficult to fill the mold. When the ratio increased to 4:1, the mixture could not be poured into the mold. Thus, the ratio of 1:1 was chosen in subsequent experiments.

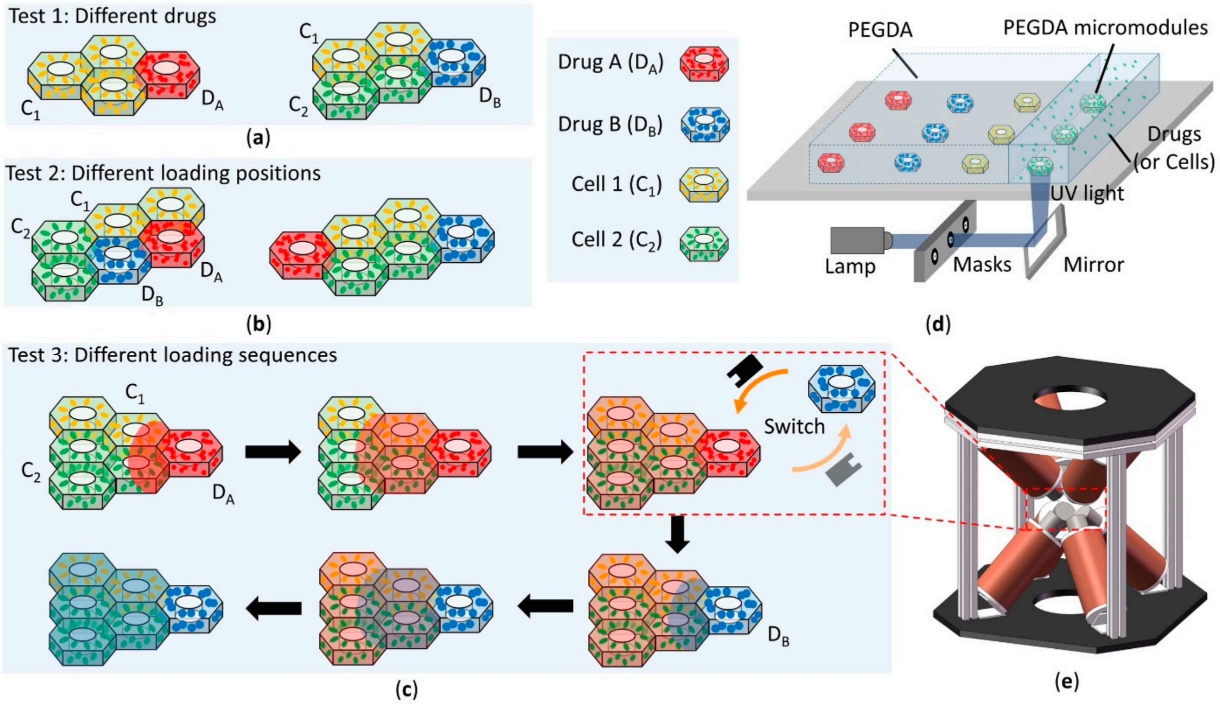

Figure S2. Schematic of proof-of-concept experiment for drug trials. (**a**) Test 1: the influence of different types of drugs to the microtissue. (**b**) Test 2: the influence of different drug loading positions to the microtissue. (**c**) Test 3: the influence of the different drug loading sequences to the microtissue. (**d**) Fabrication of different drug or cell micromodules. (**e**) The eight-coil electromagnetic system for magnetic manipulation.

The manuscript demonstrates a method to assemble different cell-laden micromodules and load different drugs, indicating huge potential for drug trials. The proof-of-concept experiment is clarified as follows. Drug trials were classified into three types as: (1) the influence of different types of drugs to the microtissue; (2) the influence of the different drug loading positions to the microtissue; (3) the influence of the different drug loading sequences to the microtissue. As shown in the Figure S1, in type (1), cells were cultured or co-cultured into different cellular models. Then, micromodules loaded with drug A and drug B were respectively assembled to the cellular models for evaluation of the influence caused by drug types. In type (2), the magnetic micromachine simultaneously assembled the micromodules with drug A and B to the different local area around the cellular model. It tested the spreading of the drugs and how they influenced cell behavior in different places. In type (3), the magnetic micromachine firstly assembled one type of the drug to the cellular model and cultured for a while. Then, the assembled drug was removed and a new drug was assembled to the same cellular model and cultured again. In this way, the influence of the drug action time to the cells was evaluated.
